# Anti-Inflammatory and Antioxidant Properties of Peptides Released from β-Lactoglobulin by High Hydrostatic Pressure-Assisted Enzymatic Hydrolysis

**DOI:** 10.3390/molecules22060949

**Published:** 2017-06-07

**Authors:** Fatemeh Bamdad, Seonghee Bark, Chul Hee Kwon, Joo-Won Suh, Hoon Sunwoo

**Affiliations:** 1 Centre for Pharmacy & Health Research, Faculty of Pharmacy and Pharmaceutical Sciences, University of Alberta, 11361-87 Ave, Edmonton, AB T6G 2E1, Canada; bamdad@ualberta.ca (F.B.); bark@ualberta.ca (S.B.); ckwon@ualberta.ca (C.H.K.); 2 Center for Nutraceutical and Pharmaceutical Materials, Myongji University, Yongin, Gyeonggi 449-728, Korea

**Keywords:** β-lactoglobulin, high hydrostatic pressure, enzymatic hydrolysis, degree of hydrolysis, antioxidant, anti-inflammation

## Abstract

Background: β-lactoglobulin hydrolysates (BLGH) have shown antioxidant, antihypertensive, antimicrobial, and opioid activity. In the current study, an innovative combination of high hydrostatic pressure and enzymatic hydrolysis (HHP–EH) was used to increase the yield of short bioactive peptides, and evaluate the anti-inflammatory and antioxidant properties of the BLGH produced by the HHP–EH process. Method: BLG was enzymatically hydrolyzed by different proteases at an enzyme-to-substrate ratio of 1:100 under HHP (100 MPa) and compared with hydrolysates obtained under atmospheric pressure (AP-EH at 0.1 MPa). The degree of hydrolysis (DH), molecular weight distribution, and the antioxidant and anti-inflammatory properties of hydrolysates in chemical and cellular models were evaluated. Results: BLGH obtained under HHP–EH showed higher DH than the hydrolysates obtained under AP-EH. Free radical scavenging and the reducing capacity were also significantly stronger in HHP-BLGH compared to AP-BLGH. The BLGH produced by alcalase (Alc) (BLG-Alc) showed significantly higher antioxidant properties among the six enzymes examined in this study. The anti-inflammatory properties of BLG-HHP-Alc were observed in lipopolysaccharide-stimulated macrophage cells by a lower level of nitric oxide production and the suppression of the synthesis of pro-inflammatory cytokines. Peptide sequencing revealed that 38% of the amino acids in BLG-HHP-Alc are hydrophobic and aromatic residues, which contribute to its anti-inflammatory properties. Conclusions: Enzymatic hydrolysis of BLG under HHP produces a higher yield of short bioactive peptides with potential antioxidant and anti-inflammatory effects.

## 1. Introduction

Whey is a byproduct of cheese manufacturing, with a broad range of applications in the food and nutraceutical industries [1]. The main protein constituents of whey are β-lactoglobulin (BLG), α-lactalbumin, and to a lesser extent immunoglobulin and bovine serum albumin [2,3]. Over the last decade, extensive studies have revealed the nutritional and functional properties of whey proteins [3].

Although whey proteins have nutritional and functional benefits, their utilization is restricted mainly due to the Cow’s Milk Protein Allergy (CMPA), which is the most prevalent food allergy, particularly in infants [4]. BLG is known as the main allergen in cow’s milk [5]. Among different techniques for reducing protein allergenicity, enzymatic digestion can be considered the most efficient. Heating or other denaturation treatments may expose new allergenic epitopes that were previously hidden in the inner protein core [6]. Enzymatic hydrolysis, however, cleaves peptide bonds and irreversibly destroys the allergenic epitopes. It was reported that BLG pretreated with high pressure and subjected to enzymatic digestion produces shorter peptides with low immunoglobulin binding and increased digestibility, which may result in reduced allergenicity [7]. Additionally, BLG hydrolysates have shown antioxidant [8,9], antihypertensive [10], immunomodulatory [11], antimicrobial [12,13], and opioid activities [14]. In a recent study, Tulipano et al. showed that BLG hydrolysates can inhibit dipeptidyl peptidase-4 (DPP-IV) activity, which is the enzyme responsible for the inactivation of incretin hormones. The incretin hormones stimulate insulin secretion that leads to a decrease in blood glucose level [15]. Hydrolysis under extreme pH and temperature increases the degree of hydrolysis and generates shorter peptides. However, undesirable and uncontrolled chemical reactions can cause side chain modification, which suppresses peptide bioactivity [16]. An efficient hydrolysis method is required to produce less allergenic short peptides without compromising their bioactive functions. Some pre-treatments such as thermal [17], ultrasound [18], and microwave [19] processes have been employed to improve the hydrolysis degree of different proteins. Several studies have also reported that pre-treatment with extremely high pressures (400–800 MPa) before enzyme hydrolysis resulted in unsatisfactory protein denaturation [7,20].

A major challenge in protein hydrolysis is the cost of the enzyme used. The simultaneous use of high hydrostatic pressure (HHP) and enzymatic hydrolysis (EH, HHP–EH) improves the activity of proteolytic enzymes and eliminates the need for pre-treatments, resulting in a lower processing cost [20]. Furthermore, there is a reduced risk of microbial growth during a reaction under high pressure, and the retention of flavour, colour, and nutritional value improves the shelf-life stability of commercial products [20,21,22].

The main objective of this research was to optimize the HHP–EH process to produce BLG hydrolysates with antioxidant and anti-inflammatory properties. Various enzymes, and pressures were used to determine the optimal conditions for BLG hydrolysis with the highest degree of hydrolysis. Enzymatic hydrolysis was also performed at atmospheric pressure (AP) for comparison. The BLG hydrolysates were then tested for antioxidant and anti-inflammatory properties, as preliminary tests for potential bioactivity. The amino acid sequences of the peptides in effective BLG hydrolysates were also identified by liquid chromatography with tandem mass spectrometry (LC-MS/MS), to determine the effective amino acids contributing to the antioxidant and anti-inflammatory properties.

## 2. Results

### 2.1. Degree of Hydrolysis (DH)

**Figure 1:**
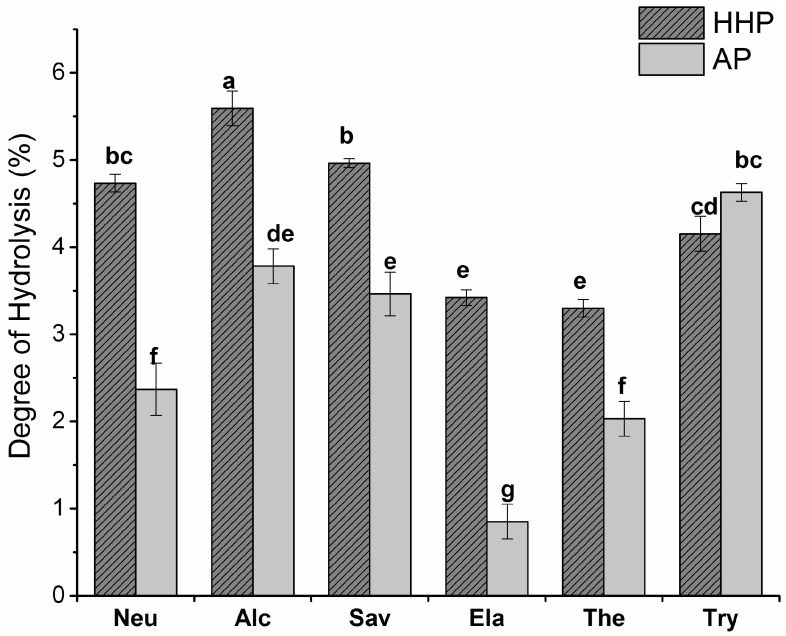
The degree of hydrolysis of β-lactoglobulin (BLG) hydrolyzed under atmospheric (AP) and high hydrostatic pressure (HHP) at an enzyme-to-substrate (E:S) ratio of 1:100. The statistical analysis of the results was performed by two-way ANOVA followed by a Duncan multiple comparisons test. The bars and error bars represent means and standard errors, respectively (*n* ≥ 3). Means with different lowercase letters differ significantly (*p* < 0.05).

The BLG was hydrolysed by neutrase (Neu), alcalase (Alc), savinase (Sav), elastase (Ela), thermolysin (Ther), and trypsin (Try), at an enzyme-to-substrate (E:S) ratio of 1:100, and under the optimal conditions for each enzyme (Table 1). The degree of hydrolysis (DH) of the BLG samples was determined by measuring the free amino group content of BLG hydrolysates treated under atmospheric and high hydrostatic pressure (Figure 1). All the enzyme treatments (except Try) displayed higher DH values under HHP. This indicates that the enzyme activity and DH is significantly enhanced by HHP. A higher efficiency of β-amylase and cellulase was reported in the extraction of saponins from ginseng roots under 100 MPa (a 1.4 and 1.5-fold increase in saponin yield, respectively), when compared to the AP condition [22].

### 2.2. Sodium Dodecyl Sulfate Gel Electrophoresis (SDS-PAGE)

**Figure 2:**
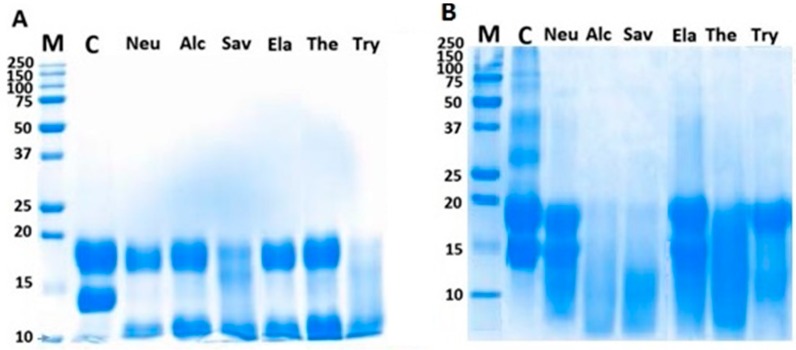
SDS-PAGE on 12% polyacrylamide Tris-Tricine gels. BLG hydrolysates were produced under atmospheric (**A**) and high hydrostatic pressure (**B**) with various enzymes (neutrase (Neu), alcalase (Alc), savinase (Sav), elastase (Ela), thermolysin (Ther), and trypsin (Try); ***M***, standard marker; C, unhydrolyzed β-lactoglobulin (BLG).

Figure 2 illustrates that the sodium dodecyl sulfate gel electrophoresis (SDS-PAGE) patterns of BLG hydrolysates treated under HHP and AP are different among proteolytic enzymes. The purified BLG before hydrolysis (lane C) showed two bands at 18 and 14 kDa, corresponding to β-lactoglobulin and α-lactalbumin, respectively. Under the AP condition (Figure 2A), the BLG showed resistance to enzymatic hydrolysis by Neu, Alc, Ela, and Ther. It was only partially hydrolyzed by Sav and Try. In HHP-hydrolysates (Figure 2B), the BLG showed less resistance, as it was almost completely digested by Alc, Sav, and Ther. Similar results were shown previously with the high-pressure-assisted proteolysis of lentil proteins. They observed a significant increase in the short peptide content of hydrolysates obtained by Alcalase, Protamex, Savinase, and Corolase 7089 [23].

### 2.3. Matrix-Assisted Laser Desorption/Ionization Time-Of-Flight (MALDI-TOF) Analysis

The MALDI-TOF spectra of BLG hydrolysates displayed a wide molecular weight range from <500 to 3000 Da. Table 2 summarizes that BLG hydrolysates under HHP showed a significantly higher content of short peptide (<500 and 500–1500 Da) than those under AP. Among the enzymes, the Alc- and Ther-treated samples contained larger proportions of very short peptide (<500 Da), and the Sav-treated sample showed the highest content of medium and large peptides (500–1500 and 1500–3000 Da), indicating the high activity of the Alc and Ther under HHP.

### 2.4. Antioxidant Capacity of BLG Hydrolysates

#### 2.4.1. 1,1-Diphenyl-2-Picryl Hydrazyl (DPPH) Scavenging Capacity

**Figure 3:**
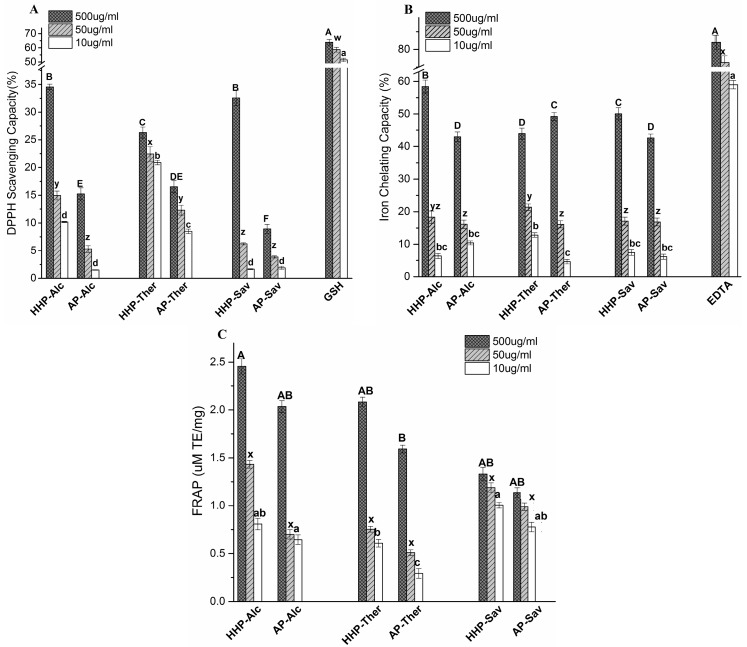
The antioxidant capacity of selected β-lactoglobulin hydrolysates produced under HHP and AP condition. (**A**) DPPH scavenging capacity; (**B**) iron chelation capacity and (**C**) ferric reducing antioxidant power (FRAP) values of β-lactoglobulin hydrolysates produced by Alcalase (Alc), Thermolysin (Ther), and Savinase (Sav). The statistical analysis of the results was performed by two-way ANOVA followed by a Duncan multiple comparisons test. The bars and error bars represent means and standard errors, respectively (*n* ≥ 3). For each concentration level, means with different lowercase letters differ significantly (*p* < 0.05).

Figure 3 illustrates a dose-dependent DPPH scavenging capacity, indicating that the BLG hydrolysates exhibited a higher scavenging capacity under HHP than under AP (Figure 3A). The result can be explained by the smaller size of hydrolysates that increases the solubility and diffusivity of peptides to interact with free radicals [24]. The BLG hydrolysates produced by Alc showed the highest DPPH scavenging capacity. It can be explained by the specificity of Alc to break peptide bonds that involve aromatic residues [25]. It leaves peptides with more exposed hydrophobic residues, which facilitates their interaction with DPPH as a hydrophobic free radical. Glutathione is a well-known oxidant scavenger which was used as a positive control.

#### 2.4.2. Iron Chelating Activity

Regardless of the pressure treatment, the BLG hydrolysates showed an iron chelating capacity (42–58% at 500 μg/mL) comparable to EDTA (58% at 10 μg/mL) (Figure 3B) in a dose-dependent manner. Hydrolysis under HHP improved the iron chelating capacity of the Alc and Sav samples (35% and 16% increase, respectively), but Ther-hydrolysates displayed lower chelating capacity after HHP-hydrolysis (12% decrease).

#### 2.4.3. The Ferric Reducing Antioxidant Power (FRAP) Assay

The reducing capacity of BLG hydrolysates was determined by FRAP assay, and expressed as a μM Trolox equivalent (Figure 3C). All the hydrolysates treated by the HHP condition showed higher FRAP values, indicating a greater reducing power due to the generation of shorter peptides. The higher FRAP values of Alc and Ther samples compared to that of the Sav sample confirmed the effect of peptide size on reducing power.

### 2.5. Anti-Inflammatory Properties of BLG Hydrolysates

The BLG hydrolysates produced by Alc resulted in the highest degree of hydrolysis, and showed the best antioxidant properties among the enzymes examined in this study. Therefore, Alc was used to determine the effect of HHP and AP on the anti-inflammatory properties of BLG.

#### 2.5.1. Cell Viability Assay

**Figure 4:**
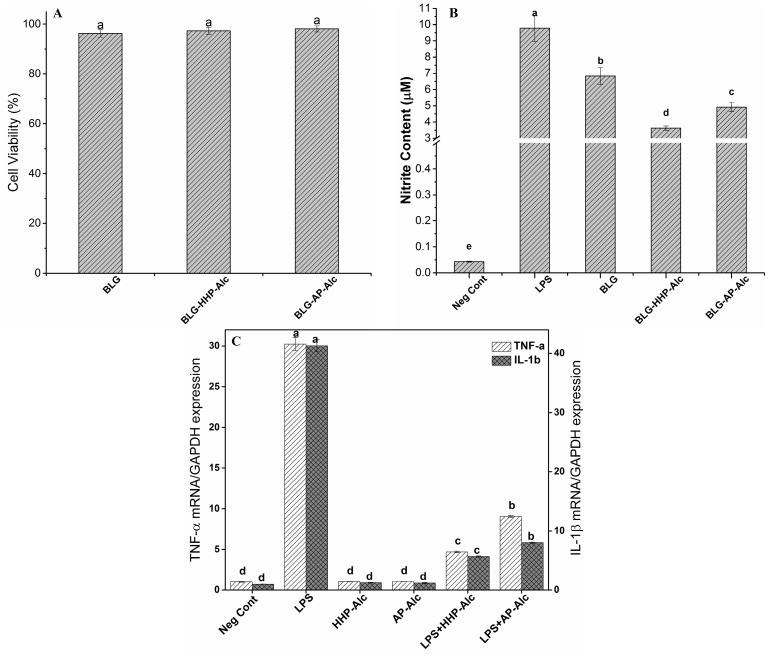
(**A**) Viability of RAW 264.7 cells; (**B**) Nitric oxide content of RAW 264.7 cell supernatant and (**C**) mRNA expression of pro-inflammatory cytokines (tumor necrosis factor (TNF-α) and IL-1β) relative to glyceraldehyde 3-phosphate dehydrogenase (GAPDH) (∆∆C_t_) in lipopolysaccharide (LPS)-stimulated RAW264.7 macrophage cells in the presence of intact β-lactoglobulin (BLG) and BLG hydrolysates produced by alcalase under HHP and AP conditions (BLG-HHP-Alc and BLG-AP-Alc). The Negative Control indicates the cells grown without any treatment. The Positive Control indicates the cells treated with LPS, without treatment. The bars and error bars represent means and standard errors, respectively (*n* ≥ 3). Means with different lowercase letters differ significantly (*p* < 0.05).

The results obtained from 3-(4,5-dimethylthiazol-2-yl)-2,5-diphenyltetrazolium bromide (MTT) assay, as presented in Figure 4A, showed that the cell viability of RAW264.7 macrophage cells was not affected by the pressure treatment (Figure 4A). The macrophage cells displayed more than 90% cell viability in the presence of BLG hydrolysates (at 1 mg/mL). It was demonstrated previously that BLG hydrolysates produced under high pressure are well tolerated by epithelial cells [26].

#### 2.5.2. Determination of Nitric Oxide (NO) Production by Macrophage Cells

Figure 4B illustrates that all the BLG hydrolysates reduced the NO levels in the macrophage cells. A negative control showed a minimal content of NO (0.04 μM NO in cell supernatant), while a positive control, which was induced by bacterial lipopolysaccharide (LPS), produced 9.8 μM NO. The production of NO was significantly lowered in the cells incubated with HHP-BLG-Alc hydrolysates (3.6 μM) compared to the cells incubated with AP-BLG-Alc hydrolysates (4.9 μM, Figure 4B).

#### 2.5.3. Gene Expression of Pro-Inflammatory Cytokines in LPS-Stimulated Macrophages

The gene expression of pro-inflammatory cytokines (tumor necrosis factor (TNF-α) and interleukin-1β (IL-1β)) in LPS-stimulated RAW264.7 macrophage cells was studied using BLG-Alc hydrolysates produced under HHP and AP conditions. The BLG-Alc hydrolysate significantly suppressed the gene expression of pro-inflammatory cytokines (TNF-α and IL-1β) in LPS-stimulated RAW264.7 macrophage cells (Figure 4C), and showed no effect on cells without LPS stimulation (negative control). This reduction of gene expression was greater with the hydrolysates produced under HHP as compared to the hydrolysate produced under atmospheric pressure, indicating a positive effect of HHP on improved anti-inflammatory properties.

### 2.6. Peptide Sequencing of the Most Effective BLG Hydrolysate

The BLG hydrolysate produced by Alc was subjected to peptide sequencing using liquid chromatography with tandem mass spectrometry (LC-MS/MS) and the results are shown in Table 3. A total of eight peptides with 6–13 amino acids were sequenced. All of them had either Leu or Phe or both at the C-terminus. Also, the amino acids with polar side chains—Tyr and Thr—occurred in the sequence of most of the peptides.

## 3. Discussion

In the last decades, whey proteins have been used to produce high quality protein hydrolysates with improved bioavailability, bioactive functionality, and lower allergenicity. Previous studies have reported the effects of different treatments on whey protein hydrolysis [18,19,27]. In this study, the innovative HHP–EH method was used to digest the major protein of whey—BLG—under mild conditions. It was shown that the extent of hydrolysis under HHP was greater than that under AP (Figure 1). A pressure of 100 MPa increased the activity of α- and β-amylases from barley malt by 25% and 16%, respectively, and enhanced the catalytic activity of α-chymotrypsin by sevenfold [28]. Similar reports also confirmed the higher efficiency of enzymes under HHP in hydrolyzing antler protein [29], and the extraction of saponins from ginseng root [22]. Stapelfeldt et al. reported that the hydrolysis of BLG by pepsin—which is a slow process under ambient conditions—was facilitated under high hydrostatic pressure (300 MPa). They also observed an enhanced hydrolysis of BLG by trypsin and thermolysin under high pressure, which was traced to BLG denaturation under high pressure [30].

Higher pressure levels—up to 100 MPa—alter the protein volume (compressibility) and hydration shell surrounding the protein molecule by changing the exposure of functional groups, leading to the higher susceptibility of protein to enzymatic catalysis [7,22,31,32]. High pressure also increases the efficiency of proteolytic enzymes by the weakening or dissociation of the stabilizing molecular interactions in the protein structure, such as hydrophobic interactions [33]. The dissociation of electrostatic bridges increases the number of charged side chains, and attracts water molecules into protein cavities. This leads to a higher rate of mass transfer, and more efficient enzyme–substrate contact [34].

The SDS-PAGE pattern (Figure 2) of hydrolyzed BLG by different enzymes under HHP showed that the protein is digested to smaller Mw peptides. This was also confirmed in the MALDI-TOF results by the increase in the shorter peptides content of hydrolysates produced under HHP.

Compared to AP-hydrolysates, the free radical scavenging and reducing capacities of HHP-BLG hydrolysates were significantly stronger (Figure 3). However, both HHP- and AP-hydrolysates showed superior iron chelating capacity (42–58% at 500 μg/mL), which was dose-dependent. Metal ion chelation is an important antioxidant mechanism, performed by different amino acid side chains of antioxidant peptides via electrostatic and coordination bindings [35]. These results suggest that the enzymatic hydrolysis caused a remarkable increase in exposed amino acid residues capable of binding metal ions (e.g., histidine and cysteine).

The MTT assay was used to demonstrate the effect of BLG hydrolysates on RAW264.7 macrophage cell viability. After a 24 h incubation, both BLG-HHP-Alc and BLG-AP-Alc showed no significant variation in cell viability (Figure 4A). The detailed mechanisms of peptide uptake and the effect of peptides on cellular metabolism require further investigation, to ensure the safe use of peptides.

Nitric oxide (NO) production is an inflammatory response to oxidative stress in cells. The overproduction of NO has been found to play a role in certain diseases, such as diabetes mellitus, neurodegenerative disorders, and other inflammatory conditions. Antioxidant peptides can suppress oxidative signaling, and therefore alleviate inflammatory responses. The LPS-stimulated macrophage cells produced NO to a degree several times higher than that of untreated cells. We evaluated the NO production of the LPS-stimulated macrophage cells in the presence of HHP- and AP-BLG hydrolysates. Cells incubated with BLG-HHP-Alc produced significantly lower NO compared to cells incubated with BLG-AP-Alc (Figure 4B). This reflects the positive effect of HHP on producing hydrolysates with anti-inflammatory properties compared to AP hydrolysis.

Different mechanisms have been proposed for the inhibition of LPS-induced effects on macrophage cell lines by proteins and peptides [36]. These mechanisms include the binding to lipid A moiety of LPS, and the interference with LPS–CD14 interaction by competition with LPS-binding protein [37]. Other factors, such as the amino acid sequence of the peptides, also influence peptide internalization by cells which may change their inhibitory effect.

In addition to NO, LPS-stimulated RAW264.7 macrophage cells can release large amounts of pro-inflammatory cytokines, such as TNF-α and IL-1β. These cytokines can further activate macrophage cells, and stimulate the production of other inflammatory cytokines, contributing to various pathophysiological conditions [38]. Downregulation of these cytokines is an important step in anti-inflammatory therapy. The BLG-Alc hydrolysate significantly suppressed the gene expression of cytokines TNF-α and IL-1β in LPS-stimulated macrophage cells (Figure 4C), and the reduction was greater with the BLG-Alc hydrolysates produced under HHP. As shown in this study, the downregulation of NO and the pro-inflammatory cytokines TNF-α and IL-1β indicate a potential anti-inflammatory property of BLG-derived peptides. Kiewiet et al. also reported the anti-inflammatory effects of BLG trypsin hydrolysates; they observed an upregulation of the anti-inflammatory cytokine IL-10 production in splenocytes obtained from mice treated with BLG trypsin hydrolysates [39].

The antioxidant and anti-inflammatory peptides from the BLG-Alc hydrolysate were subjected to LC-MS/MS analysis. Eight peptides derived from BLG were characterized. The bioactivity of peptides can be influenced by various factors, including peptide size, composition, and the positioning of the amino acids in the sequence [40]. The occurrence of terminal Leu (L) or Phe (F)—especially C-terminal Leu—has been reported to positively contribute to the antioxidant activity of peptides [41,42]. Six of the peptides characterized in this study contained Leu in the C-terminus, and the other two peptides have Phe in their C-terminus. In addition, all of the peptides are composed of aromatic residues, such as Ser (S), Tyr (Y), and Thr (T), which may contribute toward antioxidant properties. The anti-inflammatory properties of the peptides can be influenced by the presence of hydrophobic amino acid residues, such as Phe (F), Tyr (Y), Pro (P), Val (V), Ile (I), and Leu (L); and also the positively charged amino acids Arg (R), His (H), and Lys (K), in the sequence [43]. About 38% of the total amino acids of the identified peptides are composed of hydrophobic and aromatic residues, which possibly contribute to anti-inflammatory properties. A similar result was also found in the relative abundance of proline, valine, and leucine in the casein hydrolysates treated by HHP [44].

Therefore, our findings showed that HHP–EH was able to produce short peptides from BLG protein with a high antioxidant and anti-inflammatory activity. While extreme physical and chemical conditions increase the DH, these harsh conditions reduce the biological activity of the peptides. Thus, HHP–EH improves hydrolysis efficiency without compromising the peptides’ bioactivity. Furthermore, HHP–EH is a very simple and economic alternative to the conventional methods of protein hydrolysis, with a lower risk of contamination and a higher nutritional value of the product.

## 4. Materials and Methods

### 4.1. Materials

The β-lactoglobulin (85%) was purchased from Sigma-Aldrich Inc (Oakville, ON, Canada). The enzymes (Neutrase (Neu), Alcalase (Alc), Savinase (Sav), Elastase (Ela), Thermolysin (Ther), and Trypsin (Try)) were purchased from Sigma-Aldrich, MP Biomedicals (Santa Ana, CA, USA), Novozymes (Ottawa, ON, Canada), and Fisher Scientific (Burlington, ON, Canada). The mouse macrophage cell line RAW 264.7 was obtained from American Type Culture Collection (Rockville, MD, USA), and cultured in DMEM supplemented with 10% Fetal Bovine Serum (FBS), penicillin/streptomycin/fungizone, and other required ingredients. The cell cultures were maintained at 37 °C in a humidified atmosphere of 5% CO_2_. All the chemicals and reagents were of analytical grade, and obtained from commercial sources.

### 4.2. Apparatus

A potable high-hydrostatic pressure machine (TFS-2L, Toyo-Koatsu Co., Ltd., Hiroshima, Japan) was used for the HHP process, under 100 ± 2 MPa pressures at 37 °C. The vessel was filled with ultrapure water as a fluid of low compressibility. The protein–enzyme mixture, placed in a sealed pouch, was kept in a water bath to maintain a constant vessel temperature during the high hydrostatic pressure treatment.

### 4.3. Enzymatic Hydrolysis

The β-lactoglobulin was dissolved in an appropriate buffer, at the protein concentration of 10 mg/mL. The BLG solution was hydrolyzed using six different enzymes at an enzyme-to-substrate (E:S) ratio of 1:100, under HHP at 100 MPa for 2 h at 37 °C. For comparison, hydrolysis was also performed under atmospheric pressure (AP, 0.1 MPa) for 2 h at 37 °C. The above procedures were repeated in the absence of enzymes, to act as experimental controls. After the digestion, the enzymes were inactivated by heat treatment (100 °C, 10 min), and centrifuged at (4000× *g*, 4 °C, 30 min) to remove the insoluble parts. The supernatants and pellets were separated and stored at −20 °C prior to analysis. The protein content of the BLG hydrolysates was determined using the Bio Rad Proteins Assay (Bio-Rad, Madrid, Spain). The results were expressed as mg protein/g dry powder.

### 4.4. Degree of Hydrolysis

The degree of hydrolysis was determined by the quantification of the free amino groups released during the hydrolysis reaction by the *o-*phthaldialdehyde (OPA) fluorometric assay [45]. Briefly, 200 µL of OPA solution was mixed with 20 µL of each control, standard, and samples within the wells of a black microplate. After 5 min incubation at room temperature with shaking, the fluorescence intensity was measured using a Synergy H1 Multi-Mode Reader (BioTek, Winooski, VT, USA) at excitation and emission wavelengths of 350 and 450 nm, respectively. The readings were corrected using unhydrolyzed BLG to eliminate the effect of terminal amino groups. Deionized water was used as the control, and L-serine was used as the standard. The DH was calculated by the following equation:

DH = (h/h_tot_) × 100 (1)

where hydrolysis equivalents (h) is the amount of free amino groups produced during the hydrolysis, expressed as millimole serine equivalents per gram of protein (mmol/g protein); and h_tot_ is the total amount of amino groups present in totally hydrolyzed BLG with 6 N HCl at 110 °C for 24 h.

### 4.5. Sodium Dodecyl Sulfate-Poly Acrylamide Gel Electrophoresis (SDS-PAGE)

The gel electrophoresis was performed according to the method of Schaegger, in Mini-PROTEAN Tris-Trycine Precast Gels in Tris-Trycine-SDS buffer (Bio-Rad, Missisauga, ON, Canada) with 12% polyacrylamide gels [46]. The Precision Plus Dual Xtra Protein Standard (Bio-Rad, Mississauga, ON, Canada) was loaded as the molecular weight marker.

### 4.6. Matrix-Assisted Laser Desorption/Ionization Time-Of-Flight (MALDI-TOF) Analysis

MALDI-TOF-MS analysis was performed at Alberta Proteomics and Mass Spectrometry (Faculty of Medicine and Dentistry, University of Alberta, AB, Canada). One µL of each sample was mixed with 1 µL of sinapic acid (10 mg/mL in 50% acetonitrile/water + 0.1% trifluoroacetic acid), and then spotted onto a stainless steel target plate and allowed to air dry. All mass spectra were obtained using a Bruker Ultraflex MALDI-ToF/ToF (Bruker Daltonic, GmbH). The ions were analyzed in the positive mode, and an external calibration was performed by use of a standard protein mixture.

### 4.7. Antioxidant Capacity Analysis

#### 4.7.1. 1,1-Diphenyl-2-Picryl Hydrazyl (DPPH) Scavenging Assay

A DPPH free radical was used to determine the free radical scavenging activity of BLG hydrolysates at different concentrations (500, 50 and 10 μ/mL) [25]. Glutathione (GSH) was used as a positive control. The radical scavenging activity of the hydrolysates was calculated according to the following equation:

%DPPH free radical scavenging = 1 − (A_s_/A_c_) × 100 (2)

where A_s_ and A_c_ represent the absorbances of the sample and the control (everything except the protein hydrolysate), respectively.

#### 4.7.2. Iron Chelating Capacity

One mL of 20 mM FeCl_2_ was added to 0.5 mL of BLG hydrolysates (500, 50 and 10 μg/mL), and the reaction was then initiated by the addition of 1 mL ferrozine (0.5 mM). The mixture was vortexed, and left standing at 23 °C for 10 min. Ferrozine- Fe_2_Cl is a pink chromophore that absorbs strongly at 562 nm [25]. EDTA, a strong metal chelator, was used as a positive control. The ferrous ion chelating ability was calculated by the following equation:

% Ferrous ion chelating ability = 1 − (B_s_/B_c_) × 100 (3)

where B_s_ and B_c_ represent the absorbance of the sample and the control (deionised water instead of hydrolysates), respectively.

#### 4.7.3. The Ferric Reducing Antioxidant Power (FRAP) Assay

The antioxidant capacity of the samples (500, 50 and 10 μg/mL) was estimated according to the procedure described by Fogarasi et al. [47]. Trolox (1–50 µM) was used as a positive control. The results were expressed as a Trolox equivalent (TE) (µM TE/mg).

### 4.8. Cell Viability Assay

The Raw264.7 cell proliferation was evaluated using the MTT assay [26]. The cells were treated with BLG hydrolysates (1 mg/mL) diluted in media for 20 h. Then, after washing, 100 μL of MTT reagent (0.5 mg/mL final concentration) was added to each well, and incubated in the dark for 4 h. Formazan crystals were solubilized in 200 μL DMSO, and the intensity of the purple color was measured at 570 nm using a UV-visible spectrophotometer (model V-530, Jasco, CA, USA). The results were expressed as percentage viability compared to the control wells (cells grown only in media without hydrolysate treatment).

### 4.9. Determination of Nitric Oxide (NO) Production

The effect of the BLG hydrolysates on NO production by stimulated macrophages was evaluated by a determination of the accumulated nitrite (NO^2−^) in the culture media [48]. The cells were treated with BLG hydrolysates (1 mg/mL) for 4 h. After washing the cells, LPS (300 μL at 1 μg/mL) was added to each well to stimulate the macrophage cells, and incubated for 20 h. At the end of incubation time, 100 μL of cell supernatants was mixed (1:1 *v*/*v*) with Griess Reagent in 96-well plates, followed by 15 min of incubation at an ambient temperature. The absorbance was recorded at 540 nm using a UV-visible spectrophotometer (model V-530, Jasco, CA, USA). Cells grown in media, without any treatment, were taken as a negative control. The positive control cells were only stimulated with LPS (without BLG hydrolysate treatment). Sodium nitrite at different concentrations (0.1–100 μM) was used to make a standard curve (R^2^ = 0.9998).

### 4.10. Real-Time Polymerase Chain Reaction (RT-PCR) Analysis for Cytokine Gene Expression

RAW264.7 cells were seeded in a six-well plate at 5 × 10^4^ cells/cm^2^. After a 24 h incubation at 37 °C, the cells were treated with LPS (1 µg/mL) in the presence or absence of Alc-treated samples (10 µg/mL). Cells incubated in media served as the negative control. Cells treated only with LPS (1 µg/mL) were taken as the positive control. Glyceraldehyde 3-phosphate dehydrogenase (GAPDH) was used as an endogenous control. After 24 h, the RNA was extracted from the cells using the TRIzol-chloroform method. The RNA’s purity was measured by a spectrophotometric method. The total RNA was reverse-transcripted into cDNA (in 20 µL of RT-reaction mixture) using the 5X All-In-One RT MasterMix for RT-PCR. The quantitative real-time-PCR was performed in a Quantstudio III (Applied Biosystems, Carlsbad, CA, USA). The 10 µL reaction mixture consisted of 2.5 ng cDNA, 300 nM of each primer (TNF-α, forward: 5′-TACTGAACTTCGGGGTGATCGGTCC-3′, reverse: 5′-CAGCCTTGTCCCTTGAAGAGAACC-3′; IL 1-β, forward: 5′-GGAGAACCAAGCAACGACAAAATA CC-3′, reverse: 5′-TGGGGAACTCTGCAGACTCAAAC-3′; GAPDH, forward: 5′-ACTTTGTACAGCTCATTTCC-3′, reverse” 5′-TGCAGCGAACTTTATTGATG-3′), and 5 µL of EvaGreen 2X qPCR MasterMix-Low Rox. Water was used as a control. The reaction conditions were initial denaturation at 95 °C for 10 min, followed by 40 cycles at 95 °C for 15 s and 60 °C for 60 s. Each mRNA expression was normalized against GAPDH mRNA (ΔΔC_t_-method), and all data are presented as a fold change against the unstimulated control [49].

### 4.11. Liquid Chromatography-Tandem Mass Spectrometry (LC–MS/MS)

The hydrolysate was subjected to LC-MS/MS analysis on a nano UPLC (Waters, Milford, MA, USA), coupled with a q-Tof premier mass spectrometer (Waters, Milford, MA, USA). Five µL of the peptide mixture was loaded onto a nanoAcquity UPLC system, with a C_18_ PepMap 100 Nano-Precolumn™ (300 µm × 1 mm, Dionex, Sunnyvale, CA, USA), and a nano analytical column (75 µm × 150 mm, C_18_ acclaim PepMap 100 column™, Dionex, Sunnyvale, CA, USA). Desalting on the peptide trap was achieved by flushing the trap with 1% acetonitrile, and 0.1% formic acid in water at a flow rate of 10 µL/min for 1 to 3 min. The peptides were separated with a gradient of 1–60% solvent B (acetonitrile, 0.1% formic acid) over 55 min at a flow rate of 350 nL/min. The column was connected to a q-Tof premier (Waters Corporation, Milford, MA, USA) for ESI-MS/MS analysis. The obtained MS/MS data were analyzed through proteomic software called Mascot (version 2.2, Matrix science, Boston, MA, USA). The settings for the UniProt database search were as follows: parent ion and MS/MS tolerance were set to 0.1 Da and 0.2 Da, respectively, and up to two missed cleavages were selected; oxidation on methionine was selected as a variable modification. The confidence of positive protein identification was judged by high protein and peptide scores in the search results. A manual inspection of the original MS/MS spectra was often performed to make sure major peaks in the MS/MS spectra were matched and explained.

### 4.12. Statistical Analysis

All experiments were performed at least in three independent trials, and the results were reported as mean ± SD. The results were subjected to the analysis of variance using the ANOVA procedure of SAS (SAS Institute, Inc., Cary, NC, USA), and the statistical significance of differences (*p* < 0.05) was evaluated by the least significant difference test.

## 5. Conclusions

The high hydrostatic pressure enhanced the enzymatic hydrolysis of BLG by various enzymes. Therefore, a more intensive hydrolysis of BLG under high pressure resulted in a higher proportion of short peptides compared to hydrolysis under atmospheric pressure.

The Mw distribution of hydrolysates reflected in the SDS-PAGE, SEC-HPLC, and MALDI-TOF analyses confirmed a greater content of shorter peptide produced using the HHP–EH method. A large proportion of small peptides (<500 up to >3000 Da) were obtained when the BLG was hydrolysed using the HHP–EH method. These small peptides showed an excellent free radical scavenging capacity, and iron chelation and reducing power. The HHP-BLG hydrolysates were cyto-compatible, and did not adversely influence macrophage cell growth. A significant reduction in NO level after pre-incubation with BLG hydrolysates indicates the anti-inflammatory properties of hydrolysates. This was also evident by the significant decrease in the expression of pro-inflammatory cytokines after the cells were treated with HHP-BLG-Alc hydrolysate. Accordingly, HHP–EH is a promising technology to produce shorter peptides from various protein sources under a mild hydrolysis condition. The structural and functional properties of hydrolysates obtained from the HHP–EH digestion of whey proteins are currently being studied in our lab.

## Figures and Tables

**Table 1:** The enzymes applied in high hydrostatic pressure combined with enzymatic hydrolysis (HHP–EH) and atmospheric hydrolysis, and the operational conditions.

Enzyme	Sources	Proteolytic Activity ^a^	Optimum Conditions
Neutrase	Protease from *Bacillus amyloliquefaciens*	≥0.8 U/g	pH 7; 37 °C
Alcalase	Protease from *Bacillus licheniformis*	≥2.4 U/g	pH 7; 50 °C
Savinase	Protease from *Bacillus* sp.	≥16 U/g	pH 7; 55 °C
Elastase	Elastase from hog pancreas	≥4 U/mg	pH 8; 37 °C
Thermolysin	Protease from *Bacillus thermoproteolyticus*	14 U/mg	pH 7; 50 °C
Trypsin	Protease derived from porcine pancreas	30 U/g	pH 7; 37 °C

^a^ Minimum proteolytic activity of the enzyme at optimum pH and temperature.

**Table 2:** The relative area (%) of the peptide peaks obtained in the MALDI-TOF spectra of β-lactoglobulin hydrolysates produced under atmospheric and high hydrostatic pressure conditions.

Samples	Atmospheric Hydrolysis			High Hydrostatic Pressure Hydrolysis		
	<500 Da	500–1500 Da	1500–3000 Da	<500 Da	500–1500 Da	1500–3000 Da
Neu	6.88	74.38	18.75	16.18	52.21	31.60
Alc	27.24	28.65	44.11	71.15	28.38	0.47
Sav	0.16	34.84	65.00	0.26	63.44	36.30
Ela	44.42	39.02	16.55	38.66	44.70	16.64
Ther	25.45	74.55	0.00	67.06	32.94	0.00
Try	68.47	28.20	3.33	23.59	63.76	12.65

**Table 3:** The amino acid sequences of effective peptides from BLG-HHP-Alc hydrolysate identified by liquid chromatography with tandem mass spectrometry (LC-MS/MS), and their respective protein fragments in the BLG sequence found in the UniProt database (P02754).

Sequence	Ion (m/z)	Observed Mass	Calculated Mass	Source (Fragment)
GTWYSL	726.35	725.35	725.34	β-lactoglobulin (33–38)
LSFNPTQL	919.49	918.48	918.48	β-lactoglobulin (165–172)
MAASDISLL	936.47	935.46	935.46	β-lactoglobulin (40–48)
AMAASDISLL	1007.49	1006.48	1006.50	β-lactoglobulin (39–48)
DTDYKKYLLF	653.35 (2)	1304.68	1304.66	β-lactoglobulin (112–121)
IIAEKTKIPAVF	665.41 (2)	1328.81	1328.80	β-lactoglobulin (87–98)
DIQKVAGTWYSL	690.86 (2)	1379.72	1379.70	β-lactoglobulin (27–38)
ELKPTPEGDLEIL	727.39 (2)	1452.77	1452.77	β-lactoglobulin (61–73)
